# Phytochemical study of polyphenols in *Perilla Frutescens* as an antioxidant

**Published:** 2012

**Authors:** Mohammad Asif

**Affiliations:** 1 *Department of Pharmacy, GRD (PG) Institute of Management and Technology, 214- Rajpur, Dehradun, 248009 Uttarakhand, India*

**Keywords:** Anthocyanins, Antioxidants, Flavonoids, * Perilla frutescens*, Polyphenols

## Abstract

*Perilla frutescens* is an annual herb of the mint family native to East Asia. Polyphenols present in perilla have various structural varieties with large diversity of biological activities. It is direct influence the quality of perilla plant and their potential functions. Some of these products have been studied and proven to be effective source of phenolic antioxidants. The aqueous extract contains phenolic compounds such as phenolic acids, cinnamic acid derivatives, flavonoids, and lignans. Gallic acid, hydroxytyrosol (3,4-DHPEA), cinnamic acid derivatives (coumaroyl tartaric acid, caffeic acid and rosmarinic acid), flavonoids, scutellarein 7-O*-*diglucuronide, luteolin 7-O-diglucuronide, apigenin 7-O-diglucuronide, luteolin 7-O*-*glucuronide, and scutellarein 7-O-glucuronide), and anthocyanins (mainly *cis*-shisonin, shisonin, malonylshisonin and cyanidin 3-O-(E)-caffeoylglucoside-5-O-malonylglucoside) are present.

## Introduction

*Perilla frutescens *(L.) Britt. (Lamiaceae) and their varieties are edible plants frequently used in Asian countries such as China, Korea, Japan and Thailand (Asif and Kumar, 2010 ▶; Heci, 2001 ▶). Perilla is a very attractive plant for the garden and attracts butterflies. It is an aromatic plant with a strong minty smell. Growing up to four feet tall when in bloom, the stems are square, reddish-purple and branching. The leaves are large, up to 15 cm in diameter, dark green tinted red to purple, and hairy (Foster & Duke, 1990 ▶; Manandhar, 2002 ▶; Diggs et al., 1999 ▶). 

Various perilla varieties are traditionally used by local people. Leaves of *P. frutescens *are used as a vegetable. *P*. *frutescens *var. *crispa *is more often used than var. *frutescens* in China for its medicinal properties which may be differentiated by their different leaf and stem colors, which vary from green and red and to purple, indicating the existence of anthocyanins. It has been shown that the red color is mainly due to the presence of malonylshisonin (3-O-(6-O-(E)-*p*-coumaryl-β-D-glucopyranosyl)-5-O-(6-O-malonyl-β-D-glucopyranosyl)-cyanidin) (Meng et al., 2006 ▶). In the case of green-leaf chemotypes, the content of anthocyanin type compounds must be low, which in turn should affect bioactivity. *P. frutescens *is not only used as a food ingredient but also for skin creams, soaps, and medicinal preparations because of its recognized bioactivities, such as antioxidant (Ill Min, et al., 1995 ▶), anti-allergic (Makino, et al., 2003 ▶; Takano, et al., 2004 ▶), anti-inflammatory (Ueda et al., 2002 ▶), and anti-HIV-1 activity (Yamasaki, et al., 1998 ▶). The total polyphenolic contents (cinnamic, flavonic, and anthocyanic derivatives) are found in aqueous extract of *frutescens*, and *crispa *varieties (Pereira et al., 2009 ▶; Meng et al., 2009 ▶). 

*Traditional and medicinal properties*

Perilla is an edible plant and has medicinal properties. The leaves have a very pleasant sweet taste and are used as a spice, combined with fish, rice, vegetables, and soups as well as giving color and flavor to many pickled dishes. It is also chopped and combined with ginger root and salads in many Asian countries. The seeds from the plant also supplies nutritious cooking oil. The essential oil of the plant is used as a food flavoring. The entire plant is very nutritious with vitamins and minerals (Asif, & Kumar, 2010 ▶). One of the aldehyde isomers, i.e., oxime of perilla aldehyde (perillartin) is about 2000 times sweeter than sucrose. The medicinal uses of perilla as an antiasthmatic, antidote, antimicrobial, antipyretic, antiseptic, antispasmodic, antitussive, aromatic, carminative, diaphoretic, emollient, expectorant, stomachic, and tonic substance has been shown (Asif, 2011 ▶). The plant constituents confirm these properties in alternative medicine and usefulness in curing many cancers as well as various other diseases and disorders (Facciola, 1990 ▶; Huxley, 1992 ▶; Manandhar, 2002 ▶). An infusion of the plant is useful in the treatment of asthma, common cold, cough and lung afflictions, influenza prevention, nausea, vomiting, abdominal pain, constipation, food poisoning, cancers, and to restore health and balance (Takano et al., 2004 ▶; Makino et al., 2003 ▶). The stems are a traditional Chinese remedy for morning sickness. Perilla seed oil has been used in paints, varnishes, linoleum, printing ink, and for protective waterproof coatings on clothes. 

*Edible Uses*

Seedlings are added to salads, older leaves are used as a garnish or flavoring. The leaves contain about 3.1% protein, 0.8% fat, 4.1% carbohydrate, and 1.1% ash. The seeds can also be eaten cooked. Seeds from purple-leafed forms of the plant are preferred for culinary uses. The seed contains about 21.5% protein, 43.4% fat, 11.3% carbohydrate, and 4.4% ash. An edible drying oil is obtained from the seed (Manandhar 2002 ▶). It is rich in linolenic acid (polyunsaturated fatty acids; cardiotonic oil). The plant yields an essential oil which is used as food flavoring in candies and sauces (Facciola, 1990 ▶). The plant yields *0.3*-1.3% essential oil, which contains 20% citral. It is used as food flavoring and in dental products.

*Phytochemical constituents*

Several different perilla chemotypes are known. Identified constituents of perilla are apigenin, ascorbic-acid, beta-carotene, caffeic-acid, citral, dillapiol, elemicin, limonene, luteolin, myristicin, perillaldehyde, protocatechuic-acid, quercetin, rosmarinic-acid, perilla ketone, elsholzia ketone, isoegomaketone, naginata ketone, and safrole. The rosefurane, which might have potential and a cheaper substitute for rose oil in perfume. The main component is perillaldehyde and minor constituents are limonene, linalool, β-caryophyllene, l-menthol, α-pinene, perillene (2-methyl-5-(3-oxolanyl)-2-pentene), and elemicin. Perillaldehyde can cause skin allergies. This is used in the flavor and perfume industries. It also gain importance as a source of simple phenylpropanoids in the pharmaceutical industry. The high myristicin content makes this plant considerably toxic (hallucinogenic properties of myristicin). The dominating constituents of the are monoterpenoid furanes, often ketones (Pereira et al., 2009 ▶; Meng, Lozano, Gaydou, & Li, 2009 ▶). Some of these, e.g., isoegomaketone, are severe pneumotoxins that have caused fatal poisoning in cattle repeatedly. Perilla seeds contain a drying oil (40%) with high content of multiply unsaturated fatty acids (60% α-linolenic acid and 15% both linoleic and oleic acid); their medicinal value is sometimes matter of great exaggeration (Asif, & Kumar, 2010 ▶; Asif, 2011 ▶). Lastly, perilla contains the pseudotannins and antioxidants typical for the mint family. The reddish-purple color of some cultivars is caused by an anthocyanin pigment called perillanin chloride.

**General chemistry of polyphenols**

**Figure 1:**
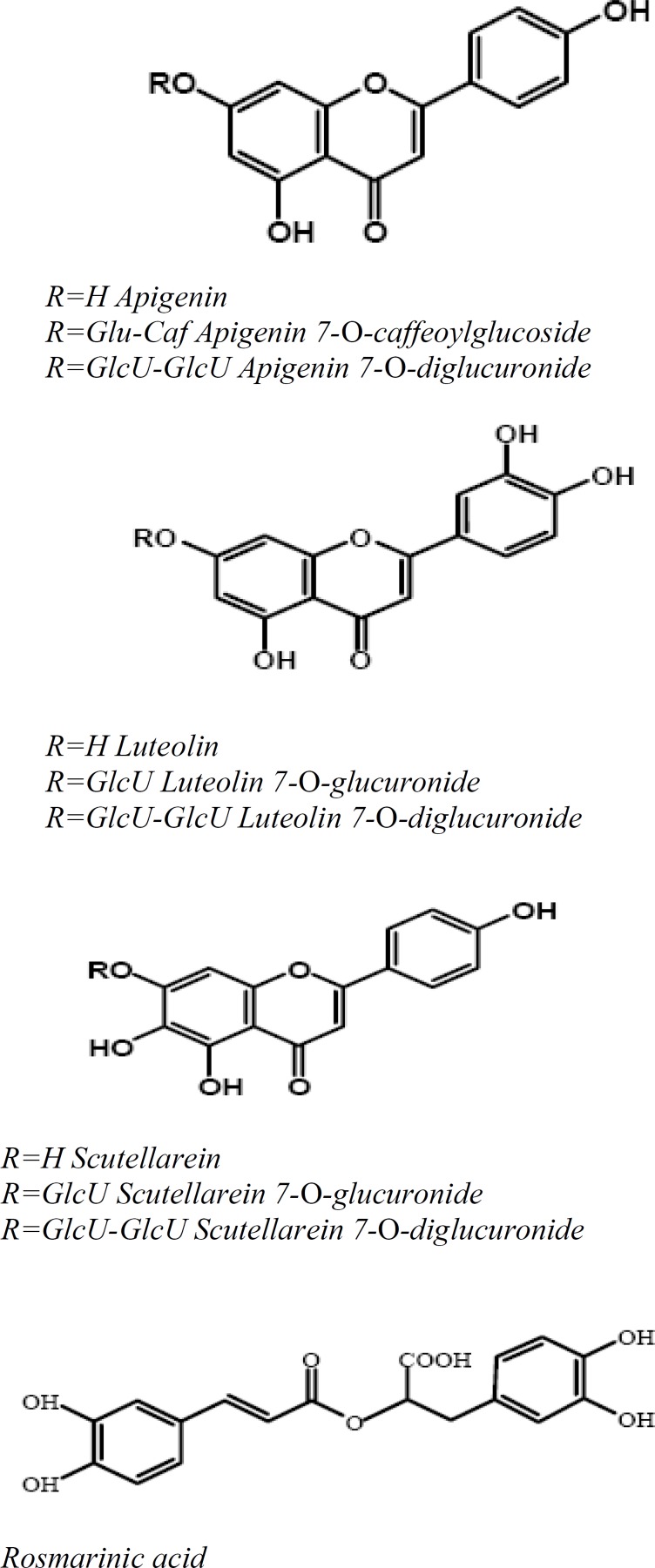
Chemical Structures of major Polyphenolic Compounds in *P. frutescens *Leaves

**Figure 2:**
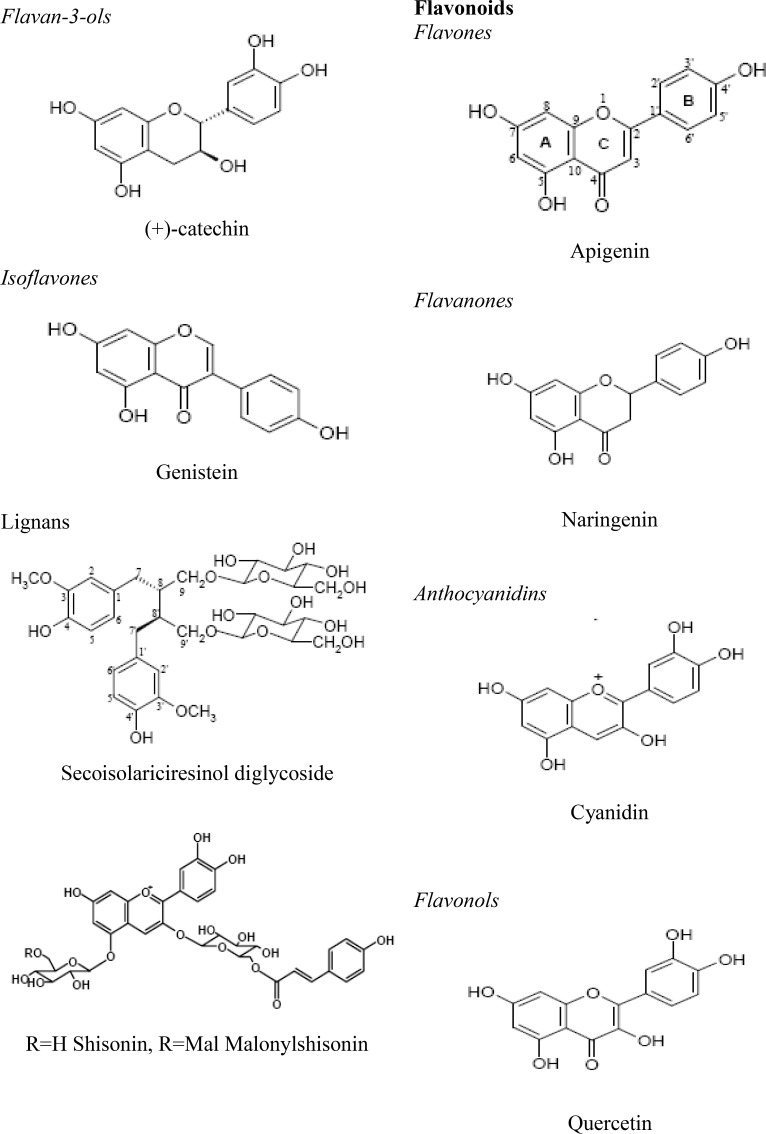
Chemical classes of polyphenolic compounds

Polyphenols are the most widespread class of metabolites in nature, and their distribution is almost ubiquitous. They generate the majority of the natural phenolics, such as flavonoids (Figure 1). Although, monophenols, such as *p*-coumaric acid, are not polyphenols, they have the same properties and characteristics as functional polyphenols (Dixon, 2004 ▶). These compounds are derived from a common carbon skeleton building block. Biosynthesis produces a large variety of plant phenols, such as cinnamic acids, benzoic acids, flavonoids, coumarins, lignans, and lignins (Seabra, et al., 2006 ▶) (Figure 2).

**Polyphenolic Compounds in** ***P. frutescens*** **Leaves**

**Phenolic acids**

*General antioxidant mechanisms of polyphenolics*

Polyphenolics are able to act as antioxidants in different ways. Phenolic hydroxyl groups are good hydrogen donors and can react with reactive oxygen and nitrogen species (Valentao, et al., 2003 ▶; Heim, Tagliaferro, & Bobilya, 2002 ▶) in a termination reaction, which breaks the generation of free radicals. The interaction with the initial reactive species results a radical form of the antioxidant which has a much greater chemical stability than the initial radical. The interaction of the hydroxyl group of phenolics with the π-electrons of the benzene ring gives the molecules special properties, most notably the ability to generate free radicals where the radical is stabilized by delocalization. The formation of these relatively long-lived radicals is able to modify radical-mediated oxidation processes (Parr & Bolwell. 2002 ▶). 

The antioxidant capacity of phenolic compounds is also attributed to their ability to chelate metal ions involved in the production of free radicals (Yang et al., 2001 ▶). However, phenolics can act as pro-oxidants by chelating metals that maintain or increase their catalytic activity or by reducing metals, thus increasing their ability to form free radicals (Croft, 1998 ▶). Phenolic structures often have the strongly interact with proteins due to their hydrophobic benzenoid rings and hydrogen-bonding potential of the phenolic hydroxyl groups. This gives phenolics the ability to act as antioxidants also by virtue of their capacity to inhibit some enzymes involved in radical generation, such as various cytochrome P450 isoforms, lipoxygenases, cyclooxygenase, and xanthine oxidase (Parr, & Bolwell, 2002 ▶). Additionally, synergistic effects of phenolics with other antioxidants, namely ascorbic acid, β-carotene, and α-tocopherol (Croft, 1998 ▶), and regulation of intracellular glutathione levels have also been described (Seabra et al., 2006 ▶).

*Flavonoids*

Flavonoids are characterized by a phenylbenzopyran chemical structure. The general structure includes a C_15_ (C_6_-C_3_-C_6_) skeleton joined to a chroman ring (benzopyran moiety). The heterocyclic benzopyran ring is known as the C ring, the fused aromatic ring as the A ring, and the phenyl constituent as the B ring. The A ring can be of two types: a phloroglucinol type that is *meta-*trihydroxylated, or a resorcinol type that is *meta*-dihydroxylated (Haslam, 1998 ▶). The B ring can be monohydroxylated, *ortho*-dihydroxylated, or vicinal-trihydroxylated. The center heterocycle most commonly exists in one of three forms: pyran, pyrilium, or γ-pyrone (Aron, & Kennedy, 2008 ▶). 

According to the position of the aromatic ring to the benzopyrane moiety, flavonoids can be grouped into four classes: major flavonoids (2-phenylbenzopyrans), isoflavonoids (3-benzopyrans), neoflavonoids (4-benzopyranes), and minor flavonoids. In plants, these compounds occur in nearly all species (Cotelle et al., 1996 ▶; Bruneton, 1999 ▶). Increasingly, flavonoids have been reported to possess many useful properties, including anti-inflammatory, oestrogenic, enzyme inhibition, antimicrobial, antiallergic, vascular, and cytotoxic antitumour activity, but the antioxidant activity (AA) is, without a doubt, the most studied one attributed to flavonoids (Meng et al., 2009 ▶). 

The well-established AA of flavonoids is also responsible for other biological activities in which the prevention of oxidative stress is beneficial. For example, the anticancer activity of some compounds is due to their ability to scavenge free radicals, thus avoiding the early stages of cancer promotion (Pereira et al., 2009 ▶). In addition to this mechanism, flavonoids have also been reported to act as anticancer agents *via *regulation of signal transduction pathways of cell growth and proliferation, suppression of oncogenes and tumor formation, induction of apoptosis, modulation of enzyme activity related to detoxification, oxidation and reduction, stimulation of the immune system and DNA repair, and regulation of hormone metabolism (Aron, & Kennedy, 2008 ▶). There are some other flavonoid classes with potent molecules that are used for the treatment of other pathologies that do not involve in the antioxidant activity. Isoflavones, whose estrogen-like capacity is now well established, are used similar to estrogen for the treatment of conditions in which the agonist effect in estrogen receptors is beneficial, such as menopause (Dixon & Ferreira, 2002 ▶). 

*Cinnamic acids*

L-Phenylalanine and L-tyrosine, as C_6_C_3_ building blocks, are precursors for a wide range of natural products. Elimination of ammonia from the side-chain to generated the appropriate *trans-*(*E*)-cinnamic acid. The phenylalanine would give cinnamic acid, whilst tyrosine could yield 4-coumaric acid (*p*-coumaric acid). All plants appear to have the ability to deaminate phenylalanine *via *phenylalanine ammonia lyase (PAL) enzyme, but the corresponding transformation of tyrosine is more restricted. The most representative cinnamic acid is caffeic acid, which occurs in fruits and vegetables mainly as an ester with quinic acid (chlorogenic acid or 5-caffeoylquinic acid) (Seabra, et al., 2006 ▶). The AA of phenolic acids is related to the number and position of hydroxyl groups in the molecule. The antioxidant efficiency of monophenols is strongly enhanced by the introduction of a second hydroxyl group at the *ortho- *or *para- *positions, and is increased by one or two methoxy substitutions in *ortho- *position with respect to the hydroxyl group (Dewick, 2002 ▶). As it happens with most polyphenols, cinnamic acids also exhibit strong antioxidant properties (Bruneton, 1999 ▶).

*Lignin and lignans*

Cinnamic acid metabolites are based on C_6_C_3_ building blocks. An important example is the plant polymer lignin, a strengthening material for the plant cell wall which acts as a matrix for cellulose microfibrils. Lignin represents a vast reservoir of aromatic materials, mainly untapped because of the difficulties associated with release of these metabolites. Lignin is formed by phenolic oxidative coupling of hydroxycinnamoyl alcohol monomers, brought about by peroxidase enzymes. The most important of these monomers are 4-hydroxycinnamoyl alcohol (*p*-coumaroyl alcohol), coniferyl alcohol, and sinapoyl alcohol, though the monomers vary according to the plant type (Dewick, 2002 ▶). Nevertheless, tyrosine inhibiting activity has been described (Azhar-ul-Haq et al., 2006 ▶). Lignans are organic compounds resultant from the establishment of a link between β-carbons of the side chain of two 1-phenylpropane derivatives. Numerous compounds possess cytostatic and antimitotic properties, perhaps their most widely known bioactivity. In addition, several other properties have been reported for lignans: inhibition of AMPc phosphodiesterase and of enzymes from the respiratory chain and antihypertensive activity (Bruneton, 1999 ▶).

*Anthocyanins*

Anthocyanins are water soluble plant pigments, usually with molecular weights ranging from 400 to 1200, and responsible for the blue, purple, and red colors of many plant tissues (Prior & Wu 2006 ▶). These compounds are glycosylated polyhydroxy- and polymethoxy-derivatives of 2-phenylbenzopyrylium (flavylium) salts. The most common sugars are glucose, galactose, rhamnose, and arabinose. These sugars are usually linked at the 3 position of the C ring or at the 5 and 7 positions of the B ring, occurring as mono-, di-, or tri-saccharide forms. Although very rare, glycosylation at the 3’, 4’, or 5’ positions of the B ring is also possible (Wu & Prior, 2005 ▶). Despite the knowledge of about 17 anthocyanidins (anthocyanin aglycones), only six of them are ubiquitously distributed in nature: cyanidin, delphinidin, petunidin, peonidin, pelargonidin, and malvidin. With the exception of 3-deoxyanthocyanidins and their derivatives, there is always a glycosyl group in C-3, which means that aglycones are rarely found in nature. The sugar moiety may be acylated by aromatic acids, mostly hydroxycinnamic acids (caffeic, ferulic, *p*-coumaric, or sinapic acids), and sometimes by aliphatic acids, namely malonic and acetic acids. These acyl moieties are usually linked to the sugar at C-3. The possibilities regarding the identity and position of sugars and acyl moieties, as well as the position and number of hydroxy and methoxy groups on the anthocyanidin skeleton, give great number of compounds, with over 600 anthocyanins (Anderson, 2002 ▶). An unusual *C*-glycosyl-anthocyanin has been isolated from the flowers of the toad lily, *Tricyrtis formosana *(Liliaceae) (Tatsuzawa et al., 2004 ▶). Recently, significant anticancer properties of some anthocyanins against a range of cell lines have been described (Zhang et al., 2008) ▶.

**Antioxidant of green and red perilla**

Leaf samples of *P. frutescens varieties *were shown different degrees of red to green colorations but the *crispa *variety is found exclusively to be in red. Water-soluble polyphenolics were extracted from the dried leaves. The red type samples, which have been identified as anthocyanins (Meng, et al., 2006 ▶). The total anthocyanin content varied from 2.9 to 4.0 μmol/100 mL (expressed as cyanidin equivalent). Malonylshisonin was the major anthocyanin in all *P. frutescens *red samples, followed by shisonin. Water extract samples showed nine compounds, including three phenolic acids (coumaroyl tartaric acid, caffeic acid, and rosmarinic acid) and six flavones (apigenin 7-O-caffeoylglucoside, scutellarein 7-O-diglucuronide, luteolin 7-O-diglucuronide, apigenin 7-O-diglucuronide, luteolin 7-O-glucuronide, and scutellarein 7-O-glucuronide). The number of phenolic compounds, other than anthocyanins, detected in both red green and red type *P. frutescens *was higher than in the green type ones. Total cinnamic derivatives content, varied from 5 to 11 μmol/100 mL and total flavones content varied from 12.0 to 18.5 μmol/100 mL in the case of red type samples (Meng et al., 2009 ▶). The AA was determined for each sample and compared with their polyphenolic compound contents. AA assays employed the inhibition of free radical 2,2- diphenyl-1-picrylhydrazyl (DPPH) test/method (Roginsky & Lissi, 2005 ▶). Leaf extract contained anthocynins (*cis*-shisonin, shisonin, malonylshisonin, and cyanidin 3-O-(E)-caffeoylglucoside-5-O-malonylglucoside), and flavones (apigenin 7-O-caffeoylglucoside, apigenin 7-O-diglucuronide, luteolin 7-O-diglucuronide, luteolin 7-O-glucuronide, scutellarein 7-O-glucuronide, and scutellarein 7-O-diglucuronide, expressed as luteolin 7-O-glucoside). The bleaching action is mainly attributed to the presence of a mixture of antioxidant compounds such as polyphenols (Meng et al., 2009 ▶).

The content of phenolic compounds and AA are partly correlated with the foliage color of the different *P. frutescens *varieties. Water extracts obtained from the red-green and red leaf color *P. frutescens *varieties contain anthocyanins, cinnamic acid derivatives, and flavonic compounds. The total polyphenols ranged from 22 up to 30 μmol/100 mL. The polyphenols in water extracts of green leaf samples show a relatively low content in total polyphenols, therefore AAs are poor for green leaf extracts. These extracts contained cinnamic acid derivatives (mainly caffeic and rosmarinic acids). The polyphenolic extraction of organic layer contained the phenolic acids, while the remaining aqueous phase contained anthocyanins and flavones. The results were expressed as rosmarinic acid equivalent for cinnamic derivatives, luteolin-7-O-glucoside for flavones derivatives, and cyanidin equivalent for anthocyanin derivatives (Meng et al., 2009 ▶).
